# Human MHC architecture and evolution: implications for disease association studies

**DOI:** 10.1111/j.1744-313X.2008.00765.x

**Published:** 2008-06

**Authors:** J A Traherne

**Affiliations:** Cambridge Institute for Medical Research, Addenbrookes Hospital Wellcome Trust/MRC Building, Cambridge, UK

## Abstract

Major histocompatibility complex (MHC) variation is a key determinant of susceptibility and resistance to a large number of infectious, autoimmune and other diseases. Identification of the MHC variants conferring susceptibility to disease is problematic, due to high levels of variation and linkage disequilibrium. Recent cataloguing and analysis of variation over the complete MHC has facilitated localization of susceptibility loci for autoimmune diseases, and provided insight into the MHC's evolution. This review considers how the unusual genetic characteristics of the MHC impact on strategies to identify variants causing, or contributing to, disease phenotypes. It also considers the MHC in relation to novel mechanisms influencing gene function and regulation, such as epistasis, epigenetics and microRNAs. These developments, along with recent technological advances, shed light on genetic association in complex disease.

## Introduction

The classical major histocompatibility complex (MHC) spans ~4 Mb and comprises over 160 protein-coding genes. Compared with other similar-sized sections of the human genome, the MHC holds the most, and some of the longest recognized associations with disease. The MHC influences numerous chronic inflammatory and autoimmune conditions, including type I diabetes, multiple sclerosis and Crohn's disease. MHC variants also confer susceptibility to many infectious diseases, such as malaria and HIV. However, for most diseases the precise genetic components and mechanisms involved remain undefined. The genetic basis of phenotypic traits within the MHC has been analysed in terms of several principle axes, including epistasis, penetrance, phenotypic plasticity, pleiotropy and polygeny. These underscore the complexity in resolving genetic effects within the MHC and relate largely to its unique evolutionary history.

The MHC, which possibly originated as a rudimentary protochordate histocompatibility locus (De Tomaso *et al*., 2005), has evolved over many millions of years to become a master coordinator of specificity in both adaptive and innate immune systems. This responsibility may explain some distinctive features of the MHC, such as high gene density and diversity, and low recombination. Yet it is these features together with the complex aetiology of multifactorial MHC-linked diseases and frequent non-Mendelian inheritance that hamper precise identification of variants contributing to disease phenotypes. In addition to being one of the most gene-dense regions of our genome, the MHC is one of the most variable. This diversity is a major contributor to individual differences in immune responsiveness.

In relation to complex diseases, the MHC's importance was recently confirmed and emphasized by whole-genome association (WGA) studies (WTCCC, 2007). The strongest associations were found with the autoimmune diseases type 1 diabetes and rheumatoid arthritis. Another WGA study demonstrated that the three major determinates for host control of HIV-1 are also contained within the MHC, serving as testimony to its further role in conferring resistance to infectious diseases (Fellay *et al*., 2007).

Many genetic risk variants in the MHC are common, suggesting that they bestow a past, or possibly enduring, evolutionary advantage. Consequently, a high proportion of individuals carry genetic risk factors for complex disease, however, the majority are healthy because of low penetrance and/or the combination of genetic and environmental risk factors required are incomplete.

Substantial evidence supports pathogen-driven balancing selection as a major force driving and maintaining human leucocyte antigen (HLA) diversity. This hypothesis is supported by a recent study which showed that populations from areas with high pathogen diversity have increased HLA diversity relative to their average genomic diversity (Prugnolle *et al*., 2005). Furthermore, other mechanisms may also be involved, such as MHC-dependent mate selection and preferential abortion (Apanius *et al*., 1997; Yamazaki & Beauchamp, 2007). Despite medical progress, pathogen-imposed selectivity must be high as infectious disease causes 50% of global mortality among those below the age of 45 (Kapp, 1999).

Characterization of HLA loci was initially motivated by their importance in transplantation. Subsequently, the typing of classical HLA genes and microsatellites, followed by variation at non-HLA candidate loci, was exploited to study MHC involvement in disease. Over time, confidence that the HLA genes act as predominant disease determinants within the MHC was established, although a small number of non-HLA genes have been identified as directly causative of some diseases. However, uncertainty still remains to which HLA genes are primarily important and to whether, or to what extent, non-HLA loci contribute to disease phenotypes.

Recently, systematic DNA sequence analyses between haplotypes have yielded information on polymorphisms and fine-structure linkage disequilibrium (LD) across the complete MHC. This information provides pools of sequence variants for disease association analysis. It also showed insight into the evolutionary dynamics and ancestral origins of common HLA loci and their haplotypes. However, there is an increasing ‘postgenomic’ appreciation of the intricacies of genome structure in modulating gene activities. Novel areas of research include chromatin modifications and microRNAs. The challenge now is to determine the role of these control mechanisms in the cellular development of a disease. This review surveys the genetic architecture of the MHC, its relationship with disease, and how it impacts on strategies to identify variants contributing to common, multifactorial disorders.

## MHC diversity

**Figure 1:**
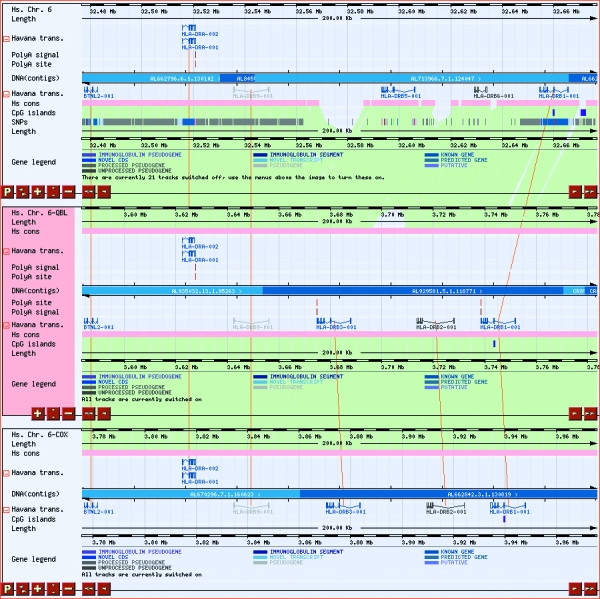
Annotation of MHC reference haplotypes in VEGA. Representation of the PGF, COX and QBL haplotypes and variation in the Vega Genome Browser ‘MultiContigView’. MultiContigView allows simultaneous display and navigation of genome annotation between the human MHC regions in the different reference haplotypes. Manually curated gene structures within the HLA-DR region are shown (see Fig. 3 and accompanying text).

With *HLA* coding variation well characterized, attention has turned to the MHC region as a whole for additional information on variability relevant to disease. Recently, four independent re-sequencing projects have significantly expanded our knowledge of variation within the MHC (Raymond *et al*., 2005; Shiina *et al*., 2006; Smith *et al*., 2006; Horton *et al*., 2008). The aims, target area, haplotype depth and strategies differed between projects. The strategies included shotgun sequencing of fosmid and bacterial artificial chromosome (BAC) libraries, as well as polymerase chain reaction (PCR)-based, direct sequencing, allowing detection of rare variants. The MHC haplotype project (Horton *et al*., 2008) determined the sequences of eight common and disease-associated MHC haplotypes. These serve as single haplotype reference sequences and can be viewed in the context of current genome annotation through the VEGA genome browser (Fig. 1).

**Figure 3:**
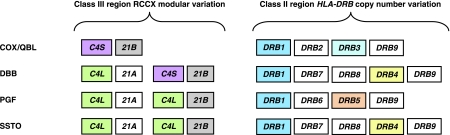
Simplified gene configurations in the RCCX and HLA-DRB regions on MHC reference haplotypes. The COX and QBL haplotype are monomodular for RCCX, whereas the PGF, SSTO and DBB haplotypes are bimodular. Each *C4* gene may encode a C4A or a C4B protein. C4L and C4S are long and short *C4* genes, respectively. 21A and 21B are *CYP21A (* pseudogene) and *CYP21B*, respectively. *DRB2*, *DRB6*, *DRB7*, *DRB8* and *DRB9* are pseudogenes.

The comprehensive catalogue of variation generated by these projects allowed the construction of high-resolution LD maps (Miretti *et al*., 2005; de Bakker *et al*., 2006b) and facilitated precise mapping of susceptibility loci for multiple sclerosis (Yeo *et al*., 2007) and type I diabetes (Nejentsev *et al*., 2007). The amassed data also draw a detailed picture of the degree and distribution of variation across the MHC as well as allowing genealogical relationships between haplotypes to be defined. Divergence between some pairs of haplotypes over parts of class I and class II can be 20-fold higher than the rest of the genome, consistent with independent haplotype evolution over tens of millions of years (assuming normal mutation rates). These extreme levels of haplotype divergence remain unprecedented in the human genome. Remarkably, human–human pairwise divergences within the MHC can be higher than human–primate comparisons (Raymond *et al*., 2005). Typically, regions of the MHC distant from classical HLA genes show the lowest levels of variation, in line with the average across the genome. However, areas of exceptional divergence (> 3%) are not limited to HLA loci, but spread over tens of thousands of base pairs into the surrounding regions. The observed broad peaks of variation may have arisen from the combined effects of balancing selection acting on the peptide-binding domains of HLA loci with hitchhiking of neighbouring neutral mutations. A novel, but untested, hypothesis proposes that some MHC-linked diseases are a consequence of hitchhiked deleterious mutations within nearby genes (Shiina *et al*., 2006). A recent study suggests that new alleles in HLA loci are being recurrently created by gene conversion (von Salome *et al*., 2007).

Regulatory regions of MHC genes may also be subject to natural selection (Liu *et al*., 2006; Loisel *et al*., 2006; Yamazaki & Beauchamp, 2007), where sequence variants conferring differential levels of expression among different cells or in different developmental stages may be the selected factors. It is predicted from recent large-scale expression analyses that variants in regulatory regions may play a greater contribution to complex disease than previously thought (Spielman *et al*., 2007). Genetic variants that modify *HLA-DR*, *HLA-DQ*, *HLA-DP*, *HLA-A* and *HLA-C* have been identified (Dixon *et al*., 2007). The strength of the observed effects implies that associations of MHC class I and II polymorphism with disease may relate to the level of gene expression as much as the restriction of response to antigen. Characterization of regulatory region architecture and transcriptional regulation of MHC genes may therefore elucidate the molecular basis for some MHC associations to diseases and particular immune responses (Eagle *et al*., 2006; Baena *et al*., 2007; Rodriguez-Rodero *et al*., 2007; Venkataraman *et al*., 2007). Moreover, other variation not associated with the peptide-binding grooves, such as that affecting the cytoplasmic tail of HLA molecules, may be critical as it alters their export to the cell surface (Boyle *et al*., 2006).

MHC genetic variation also affects alternative splicing, which expands the information content and versatility of the transcriptome through the expression of multiple different mRNAs from individual genes (Rollinger-Holzinger *et al*., 2000; Boyle *et al*., 2007; Greetham *et al*., 2007). Current annotation of the MHC reference sequence (PGF) indicates that the total number of splice variants is almost fourfold higher than the total number of loci (Horton *et al*., 2008). Some MHC loci possess over 20 splice variants, e.g. *BAT1/3* and *LST1* (VEGA). At least eight MHC loci are known to exhibit haplotypic variation at their splice sites, which may affect expression at the post-transcriptional level (Kralovicova *et al*., 2004; Traherne *et al*., 2006a; Horton *et al*., 2008). A significant proportion of sequence variation outside splice sites is also likely to affect splicing efficiency (Hull *et al*., 2007). A growing number of reports are identifying disease-susceptibility alleles, across the genome, that are associated with specific splicing patterns (Wang & Cooper, 2007). Alterations in splicing can be directly causative, modify severity or be linked with disease susceptibility. Full characterization of the MHC transcriptome and splicing code is therefore likely to provide new insights, and useful diagnostic and prognostic tools for MHC-linked diseases.

## Gene clustering

**Figure 2:**
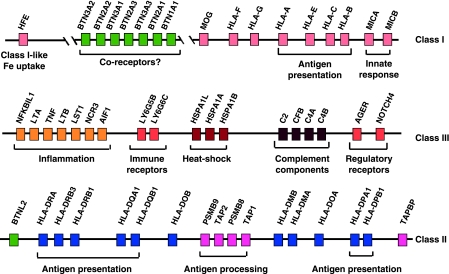
A reduced map of the MHC illustrating clustering of immune system genes. Two kinds of gene clustering are apparent. First, sequence-related duplicates that have allowed diversification of duplicates, e.g. *HLA* or *C4* complement genes. Second, sequence unrelated genes with related immune function, e.g. *PSMB8/9* — proteosome components, *TAP1/2 —* peptide transporters, *TAPBP* — peptide chaperone, and *HLA* — peptide presentation.

The MHC is one of the most gene-dense regions of the genome. One explanation for this is that the region favours high levels of expression (Horton *et al*., 2004). It contains a disproportionately high number of immune-system genes (Fig. 2). Possible advantages of clustering of immune-system genes in the MHC include enhanced coordination of gene expression and facilitated sequence exchange among sequence-related gene duplicates (paralogues). Gene families can rapidly diversify by recombination and sequence exchange, making them especially adaptable evolutionarily. A concept expanded on below proposes that clustering of polymorphic genes may help to account for marked LD within the MHC.

## Structural variation

Recent advances in global genome-wide analysis have revealed abundant and variable segmental duplications of genomic regions among different individuals, highlighting structural and interindividual copy number variation (CNV) as an important source of genetic diversity in relation to disease (Conrad *et al*., 2006; Redon *et al*., 2006; Wong *et al*., 2007). Two regions of the MHC associated with disease have long been confirmed to have gene CNVs. The first is in the class II region between *HLA-DRB1* and *HLA-DRB9 (* a non-functional gene segment) that in some haplotypes houses one additional functional *DRB* gene (*HLA-DRB3*, *HLA-DRB4* or *HLA-DRB5*), and one, two or no additional copies of *HLA-DRB* pseudogenes (*HLA-DRB2*, *HLA-DRB6*, *HLA-DRB7* or *HLA-DRB8*). The second region is the tandem duplication in the class III region termed the RCCX (*R* P-C4-CYP21-TN*X*) module, which contains a gene for complement component C4. In addition to a *C4* gene, each duplicated module includes a disrupted sequence (*CYP21A* pseudogene) for the steroid cytochrome P450 21-hydroxylase (*CYP21B*) gene (Chung *et al*., 2002). A single MHC haplotype can contain one to four copies of the RCCX module (Chung *et al*., 2002) (Fig. 3).

Sequence exchange (gene conversion) among variants of RCCX modules is a mechanism that has generated diversity and has introduced deleterious mutations with pathological consequence. For example, mutations of *CYP21A* transferred to *CYP21B* are the primary cause of congenital hyperplasia (Friaes *et al*., 2006; Lee *et al*., 2006).

Sequence duplications of related CNVs are highly homologous but generally do not share identical DNA sequence. Close examination often reveals additional genetic events that result in new polymorphisms adding new functions to existing gene products. In the case of the RCCX, the breakpoint(s) of duplication are identical among duplicated modules, but secondary genetic diversifications have led to the emergence of two forms of *C4* genes (short and long, the size difference resulting from the integration of the endogenous retrovirus HERV-K into its ninth intron) and two isotypes of C4 proteins (C4A and C4B), each with multiple allotypes.

Gene CNV can result in differential levels of gene expression and by this means could account for a significant proportion of phenotypic variation in human health and disease (Gonzalez *et al*., 2005; Aitman *et al*., 2006; Fellermann *et al*., 2006). Pertinent in this regard are the strong correlations of *C4* gene CNV with human systemic lupus erythematosus (SLE), from a recent study (Yang *et al*., 2007). A dose-dependent effect was seen; from increased SLE disease risk at low copy number, to protection against disease susceptibility at high copy number. Typical of many susceptibility genes associated with an autoimmune disease, the risk factor (low copy number of *C4*) is present in the general population, but the prevalence is significantly increased in the patient population.

In addition to the above structural variation, many polymorphisms resulting from presence/absence of retroviral sequences (e.g. Alu, LINE, HERV, LTR, MER and SVA) have been observed among MHC haplotypes. The retroviral insertions are often located either in the HLA-DR region or near the classical HLA class I genes. The significance of their targeting to HLA regions is unclear, although their presence may promote molecular evolution by facilitating non-homologous recombination or gene conversion events (Stewart *et al*., 2004; Wurtele *et al*., 2005; Sen *et al*., 2006; Zhi, 2007). Intriguingly, a recent study highlights the possibility that a HERV-derived gene, *HCP5*, located at < 100 kb from *HLA-B*, may contribute to host control of HIV-1 attributed to allele B*57 (Fellay *et al*., 2007).

## Recombination

MHC divergence patterns indicate that evolutionarily successful recombination events have been infrequent compared to the genome as a whole. Recombination fluctuates greatly across the region, but overall MHC recombination is considerably lower compared with the genome average (Miretti *et al*., 2005; The International HapMap Consortium, 2005; de Bakker *et al*., 2006b). In other words, many combinations of alleles at MHC genes rarely become separated over long periods of evolution. One interpretation is to suppose that certain MHC genes are tuned to work together, as a set of alleles on a particular haplotype (interactions between DNA sites in *cis*). Hence, the absence of detectable recombination may reflect maintenance of allele combinations with favoured immunological function through selection against unfavourable recombinant haplotypes. The MHC contains many polymorphic genes, encoding products with related functions (e.g. *PSMB8/9* — proteosome components; *TAP1/2 —* peptide transporters; *TAPBP* — peptide chaperone; *HLA* — peptide presentation) or components (e.g. *HLA-DQA* and *HLA-DQB*). It therefore seems appropriate that such genes are inherited as a coordinated set. The notion of genetic interaction (epistasis) within the MHC is expanded on below.

Although preferred combinations of alleles are conceivably the decisive determinant of haplotype selection, it may also have arisen through recombination suppression in regions of high divergence (Shen & Huang, 1989). The extent of reduced recombination rate acting on diverged haplotypes could be assessed by high-resolution recombination analysis of sperm typing. This experiment would help resolve the question of whether haplotype divergence is predominantly the effect or the cause of the prevailing patterns of allelic association. Also warranting further investigation are sequence inversions, which can be difficult to detect but may also influence LD patterns within the MHC and maintenance of extended haplotypes (long regions where multiple SNPs are perfectly correlated), as it is envisaged that recombination is impeded or may be lethal over an inversion interval between chromosomes with and without the inversion. Inverted and non-inverted copies of the region therefore evolve independently. Inversions are not uncommon in the human genome (Khaja *et al.*, 2006) and there is evidence of inversions in this region in mouse (*t* complex) so inversions could account for the strong LD in the human MHC. So far, direct evidence for inversions is lacking, but new scanning methods will assist future investigation (Bansal *et al.*, 2007; Flores *et al.*, 2007; Korbel *et al.*, 2007).

**Figure 4:**
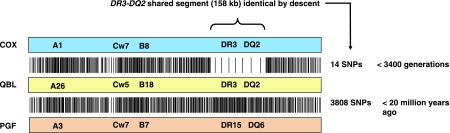
A shared ancestral block between two DR3 -DQ2 MHC haplotypes. The COX and QBL haplotypes share an ~158 kb ancestral block, which contains the *HLA-DRB2*, *HLA-DRB1*, *HLA-DQA1*, *HLA-DQB1* and *MTCO3P1* loci. Within this block there are only 14 single nucleotide polymorphisms (SNPs) distinguishing the two *DR3-DQ2* haplotypes, whereas there are 3808 SNPs that differ between the *DR3-DQ2* haplotypes and the *DR15-DQ6* haplotype (PGF). This difference can be calculated to correlate with ~3400 generations that elapsed since the two *DR3-DQ2* haplotypes separated vs. greater than 20 million years that separate the *DR15-DQ6* haplotype from the *DR3-DQ2* haplotypes. Vertical lines represent SNPs. HLA types for each haplotype are shown.

Some recombination between deeply diverged MHC haplotypes appears to have occurred relatively recently compared to the estimated divergence times of such haplotypes. Recombination of this type was recently documented in the comparison of two complete MHC haplotype sequences that are associated with different diseases (Traherne *et al.*, 2006b). The comparison showed distribution patterns of variation across the MHC that were similar to those of other HLA-disparate haplotypes, apart from a near identical sequence of about 158 kb within the class II region, consistent with relatively recent common ancestry (< 3400 generations) (Fig. 4). Such shared segments, as in this example, can be used to rule out sections of the haplotype from containing sequences that control disease specificity.

**Figure 5:**
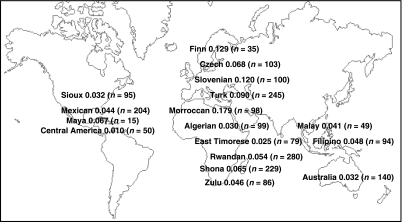
Frequencies of the DR3-DQ2 haplotype within 18 populations. The figure represents the ethnic diversity of DR3-DQ2 haplotype distribution (NCBI dbMHC).

Block swapping between haplotypes provides a footprint to trace evolution. The shared ancestral block, which encompasses the *HLA-DRB1* and *HLA-DQB1* loci (*DR3-DQ2*), is present in a number of ethnically diverse populations (Fig. 5). Similarly, other ancient, highly diverged *DR-DQ* blocks, such as *DR15-DQ6*, are also carried on diverse and widely distributed haplotypes. This suggests that numerous highly diverged, ancestral blocks are maintained in the population, and recombination enables their occasional inclusion into different haplotype backgrounds across populations, where they may be selected in response to new disease challenges. In other words, swapping of ancestral blocks is a potential mechanism, whereby certain allelic combinations with immunological advantage, in terms of HLA functions and binding specificities for example, can spread across haplotypes and populations.

Tempting as it may be to speculate that the spread of ancestral blocks by interhaplotype exchange may have been driven by selection, the frequency gradients between populations of the derivative haplotypes could conceivably be the outcome of neutral genetic drift and demographic history (Gherman *et al*., 2007). Further population studies will elucidate whether these stable ancestral sequences carry a particular selective advantage that were key to disease resistance or survival in the evolutionary history of humans.

## Long-range haplotypes

LD varies markedly along the MHC but in general LD decay over the region falls within the typical range of genome distribution (Miretti *et al*., 2005; The International HapMap Consortium, 2005; de Bakker *et al*., 2006b). However, extensive LD is evidenced by the relatively high occurrence of a handful of distinct long-range haplotypes. These haplotypes have long been of interest because of their prevalence and strong associations with complex diseases, yet the mechanisms for their generation are still unclear. Several possible explanations exist for long-range LD, including low recombination rate and polymorphic inversions. Another is maintenance of functionally coordinated sets of alleles by selection. A simpler explanation would be that strong positive selection for a component of these haplotypes has expanded their frequency in recent evolutionary history, creating founder haplotypes that have had inadequate evolutionary time to degrade.

Recent global studies using long-range haplotype (LRH) and extended haplotype homozygosity (EHH) tests support the hypothesis that signatures of positive selection are present in the MHC (The International HapMap Consortium, 2005, 2007; de Bakker *et al*., 2006b; Voight *et al*., 2006; Sabeti *et al*., 2007). Interestingly, MHC genes were not among the strongest signals identified in these genome-wide studies. This observation may reflect the nature of the MHC, where balancing selection is also active and pre-existing alleles may be recurrently selected, as well as the sensitivities of the tests; LRH tests have limited power to detect selection on standing (pre-existing) variation and EHH tests have limited ability to detect low frequency selective sweeps.

Two of the most frequent long-range MHC haplotypes in Northern Europeans are the HLA haplotypes HLA-A1-B8-C7-DR3-DQ2 (also termed AH 8.1) and HLA-A3-B7-C7-DR15-DQ6 (the so-called ancestral DR2 haplotype). The frequencies of these haplotypes in European Caucasians are in the order of 10%, which is substantially higher than expected (< 0.5%) based on the frequencies of individual alleles on the haplotypes. The high sequence similarity of long-range haplotypes suggests that their prevalence is likely due to significant expansions in relatively recent times. Indeed, the AH 8.1 haplotype has been estimated to have diverged from a single common ancestor about 23,500 years ago (Smith *et al*., 2006). In other words, the constituent alleles on these haplotypes are in strong association probably because of recent expansion rather than having been derived from distinct haplotypes through multiple independent events in which selection drove or maintained the linkage of these alleles. This view suggests that there is not necessarily any functional significance to the linkage of constituent alleles of long-range haplotypes because only one locus allele under strong positive selection in this chromosome interval could have been sufficient to drive expansion of a haplotype. The occurrence of a number of other extant haplotypes that include DR3-DQ2 but have lost HLA-A1 or HLA-B8 is consistent with this theory.

The estimated ages of some common long-range haplotypes typically fall within range of the human migration out of Africa and expansion into Europe. This has led to theories of selective factors that acted to expand these haplotypes, which in general relate to adaptation to new environments, changes in nutrition, or resistance to infection during migrations (Distante *et al*., 2004; Moalem *et al*., 2005). Although their exact anthropological origins will be difficult to determine, more precise estimates for the period and the length of time such selection was operating should be possible by comparative study and phylogenetic analysis of MHC haplotypes in different populations. The identification of the variant(s) responsible for the hypothetical selective events that led to expansions could be informative. The *DRB1* gene is a prime suspect on the DR2 haplotype (Miretti *et al*., 2005) and it is associated with susceptibility to multiple sclerosis and protection from type 1 diabetes (Barcellos *et al*., 2006; Nejentsev *et al*., 2007).

Any past survival benefits of common long-range haplotypes have come at a cost since many predispose to inflammatory diseases prevalent in Westernized society, such as type I diabetes and multiple sclerosis. The inherently strong and long-ranging correlations (extending beyond the MHC in some instances) between alleles on these haplotypes have limited the identification of haplotype-residing variants that contribute to associated disease phenotypes. Improved resolution will likely be achievable by applying higher-throughput sequencing and higher-resolution SNP analysis to define the extent of similarity between haplotypes and refine boundaries of homozygosity. In this way the evolutionary history and associations with diseases may be traceable.

## Haplotype tagging

The International HapMap Project was set up to map human variation by characterizing LD patterns across the whole genome (Thorisson *et al*., 2005; Frazer *et al*., 2007). The project is enabling the selection of informative tag SNPs that can act as surrogate markers for most common variants in human populations. This reduced set of genetic markers provides an effective, cost-efficient means to conduct genome scans in large samples and to investigate disease associations in greater detail.

The genetic complexity of the MHC necessitated a more focused project to provide a refined resource for this critical region (de Bakker *et al*., 2006b). This study characterized the LD relationships between the highly polymorphic HLA genes and background variation by typing the classical HLA genes and > 7500 common SNPs across the extended MHC region of 7.5 Mb in four population samples (African, European, Chinese and Japanese ancestry). The data add significantly to our knowledge of the underlying LD block structure. The analysis also provided informative tag SNPs that capture much of the common variation in the region to facilitate efficient fine-mapping of MHC-associated disease (de Bakker *et al*., 2006a). However, it remains to be assessed how efficiently less common variants, haplotypes or recombinants can be captured via the tagging approach. With continued advancement in sequencing technology, re-sequencing will probably continue to be a preferential method for detecting rare variants outside HLA genes that influence complex traits.

There is currently no knowledge of tag SNPs that can serve as surrogates of the RCCX CNVs. However, analysis has shown that SNPs outside the HLA genes can be informative about HLA types (de Bakker *et al*., 2006b). Hence, tags could potentially provide a simpler, complementary approach to classical HLA typing for matching transplant donors and patients or for prescreening subjects for further analysis. However, several factors make HLA tagging more challenging than tagging of SNPs. First, balancing selection has resulted in a multitude of *HLA* alleles in populations, the majority of which are not common but collectively they account for a significant proportion of the genetic diversity. Furthermore, certain forms of balancing selection, such as pathogen-driven, generate new combinations of alleles across multiple HLA genes. Consequently, a given *HLA* allele might be found on a number of different haplotype backgrounds. These effects, in addition to demography and genetic drift, mean that although tagging of certain common *HLA* alleles in some populations may be relatively straightforward, selection of tags and the boundaries of haplotype blocks are likely to differ between populations and HLA haplotypes. However, once defined, differences in LD structure between populations can be helpful in differentiating specific gene effects (Oksenberg *et al*., 2004).

## Epistasis

In the MHC, a region of the genome where at least 30% of genes are immune related and many share interrelated functions, it would be surprising if extensive epistasis were not active. This is an important consideration in disease-mapping studies within the MHC since epistasis can hinder the search for susceptibility loci; inherently the effect of one locus is altered or masked by the effects of another. However, genetic interactions can be informative by revealing not only the nature of the mutations but also gene function, functional redundancy and protein interactions. A range of statistical methods can identify or control for the presence of epistasis, although the degree to which statistical modelling can elucidate the underlying biological mechanism is limited. Consequently, the identification of true interactions can sometimes be better achieved via molecular, rather than statistical approaches.

A recent study highlights the value of molecular studies in characterizing epistatic interactions. The major candidate region for multiple sclerosis susceptibility comprises alleles of two genes located 85 kb apart that show almost complete LD in all ethnic groups studied to date, namely HLA-DRB1*1501 and HLA-DRB5*0101. The tight linkage between these two *DR* alleles might relate to the persistence of a founder haplotype, although its occurrence in diverse ethnic groups opposes this theory. A study using functional analysis in humanized mice indicates that the LD between the two alleles may be due to epistasis, whereby one allele (DRB5*0101) modulates the severity of the immune response activated by the second allele (DRB1*1501) through peripheral T-cell deletion (Gregersen *et al*., 2006). This particular epistatic interaction was found to be associated with a milder form of multiple sclerosis-like disease in mice.

Interactions of this type might prove to be a general regulatory mechanism for modifying immune responses and could be widespread throughout the MHC, contributing significantly to patterns of LD. For instance, LD in the *HLA-DQB1–HLA-DQA1* region and *HLA-DRB1* alleles could reflect the effects of persistent or strong selection for maintenance of preferred combinations of *HLA-DQB1*, *HLA-DQA1* and *HLA-DRB1* alleles. Interestingly, specific allele combinations of these three loci are major determinants of predisposition to type 1 diabetes (Koeleman *et al*., 2004).

In addition to epistasis internal to the MHC, there is mounting evidence for interactions between MHC genes and unlinked genes outside the complex resulting in susceptibility to disease. Of prominence are HLA class I interactions with the killer immunoglobulin receptor (KIR) genes encoded in the leucocyte receptor complex (LRC) on chromosome 19q13. KIRs are expressed by natural killer (NK) cells as well as subsets of T cells and interaction with their ligands, HLA class I molecules, modulates both innate and adaptive immune responses. Combinations of HLA class I and KIR variants have been associated with pathologies as diverse as autoimmunity, viral infections, pregnancy-related disorders and cancer (Parham, 2005; Khakoo & Carrington, 2006). Thus, interactions between KIR and MHC class I polymorphisms have probably been involved in human survival during incidences of epidemic infections and have affected reproduction and population expansion. These types of selection pressures might explain the functional coevolution of KIR with diverging HLA class I molecules and why KIR sequences, like the HLA loci, are highly polymorphic and rapidly evolving (Guethlein *et al*., 2007; Martin *et al*., 2007; Single *et al*., 2007).

**Figure 6:**
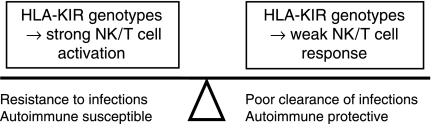
Synergy of *HLA-KIR* (human leucocyte antigen-killer immunoglobulin receptor) compound genotypes and duality between autoimmunity and infection. The general paradigm for *HLA-KIR* epistasis based on current disease association studies. Different *HLA*-*KIR* combinations provide different levels of activation and inhibition of NK (natural killer) and T cells resulting in differing susceptibility and protection against infection and autoimmunity.

Multiple factors complicate the interpretation of KIR- HLA disease association, including (i) the extensive polymorphism of the KIR and HLA class I ligands, (ii) the LD between variants within each gene family, (iii) incomplete knowledge of KIR ligands and oversimplification of the structural complexities of their interactions, and (iv) limited understanding of KIR gene expression control. Despite these complications, several points emerge from the data. In general, KIR-HLA combinations with a tendency towards stronger NK cell activation or lower levels of inhibition are associated with increased risk of autoimmune diseases but tend to be protective against infectious diseases. On the other hand, stronger inhibitory combinations are associated with protection against inflammatory diseases and disorders in pregnancy (Fig. 6). The data are consistent with the idea that disease susceptibility is modified by specific KIR-HLA ligand interactions. For this reason several studies have examined KIR and HLA class I combinations in disease association studies. Such studies will need to be large, with well-controlled populations, in order to be adequately powered. In order to rule out false positives, risk variants will likely have to undergo replication tests of validity because of the many semi-independent tests that will have to be considered. A recent study describing the specific combinations of *KIR3DL1* and *HLA-B* alleles that influence HIV disease progression exemplifies the significance of KIR-HLA epistasis in disease-resistance and the importance of distinguishing between alleles. The data also affirm a key role for NK cells in limiting HIV infection (Martin *et al*., 2007).

## Epigenetics

Epigenetic changes refer to chemical modifications of chromatin without alteration to the underlying DNA sequence itself. Combinations of these changes modulate the accessibility of regulatory DNA sequences to transcriptional activators and repressors. In this way, collective gene activation and local control of gene-specific transcription is controlled by the ‘epigenetic code’ (Bernstein *et al*., 2007). This complex regulation and interplay are believed to have an essential role in cellular differentiation, allowing cells to stably maintain different characteristics despite containing the same genomic material. It is also invoked in diverse cellular phenomena such as chromosomal stability, gene silencing and parental imprinting. Modifications of chromatin components include methylation of DNA cytosine residues and post-translational alterations to histone proteins, including acetylation, phosphorylation and ubiquination. Such changes are maintained when cells divide, and although most of these features are considered dynamic through development, some epigenetic features may display trans-generational inheritance (Crews *et al*., 2007; Jirtle & Skinner, 2007). However, since these modifications can be influenced by endogenous and exogenous factors, the epigenome is essentially the interface between genetics and the environment, where the hardwired primary genetic code is modulated by the plasticity of epigenetic code.

To date, genetic studies of MHC-linked disease have almost exclusively concentrated on DNA sequence differences. However, given that a full explanation for most MHC correlations with disease has not yet been reached, it seems rational to consider the involvement of epigenetics. Interest is heightened by several inferences; first, the association of epigenetic dysfunction in other parts of the genome with human diseases (Feinberg, 2007). Second, the fact that some MHC genes, including *HLA* genes, are already known to be regulated by epigenetic events (Wright & Ting, 2006). Also several epigenetic phenomena, such as imprinting, violate Mendelian principles.

One area of focus is epigenetic modulations on HLA and their effects on tumours. Inactivation of classical HLA genes through DNA hypermethylation may be one of the mechanisms through which cells create an immune privilege and through which neoplastic cells escape immune recognition. Conversely, transcription activation of non-classical *HLA-G* through demethylation, and resultant HLA-G protein expression, may facilitate tumour cells to evade immunosurveillance. In accordance, glioma cell lines expressing HLA-G are resistant to allo-reactive lysis (Wiendl *et al*., 2002), and HLA-G-positive melanoma cell lines are protected from NK cell cytolysis (Cabestre *et al*., 1999). Correlation between promoter methylation and *HLA-G* repression, and the observation that a demethylating treatment reverses promoter activity *in vitro*, lend credence to this hypothesis (Mouillot *et al*., 2005).

As opposed to genetic alterations, epigenetic events can be modified by pharmacological agents that induce DNA hypomethylation or inhibit deacetlylation. Consequently, there is interest to manipulate this system therapeutically, for example to generate anticancer immune responses or to dampen-down autoimmune reactions (Yoo & Jones, 2006). For example, methylation inhibitors might provide benefits in certain inflammatory diseases where HLA-G is suspected to function as an inhibitory feedback signal. However, current epigenetic treatments lack specificity. For instance, epigenetic cancer therapies using DNA methylation inhibitors to prevent inactivation of classical HLA may in turn activate expression of HLA-G and thus could favour tumour immune escape. Systematic efforts are underway to measure specific forms of epigenetic information throughout the genome. The Human Epigenome Consortium is a public-private collaboration that has initiated the Human Epigenome Project (HEP) in order to identify and catalogue methylation variable positions (MVPs) in the human genome. The MHC was chosen as the first target of the project because it is highly polymorphic, gene-rich and associated with disease. The study investigated seven cell types (adipose, brain, breast, lung, liver, prostate and muscle) across 32 individuals (Rakyan *et al*., 2004). Over 100 000 CpG methylation values within 253 amplicons were analysed. The second phase of HEP comprised about 1.9 million CpG methylation values within 2524 amplicons across chromosomes 6, 20 and 22 in 43 samples (derived from 12 different tissues) (Eckhardt *et al*., 2006). In both studies, the methylation profiling was undertaken by high-throughput bisulphite DNA sequencing. The combined data from these projects have been integrated with the human genome sequence using the ENSEMBL interface to establish a database (http://www.epigenome.org), where the findings of the MHC pilot study and the second HEP project are publicly available. Overall, the methylation profiles were similar in both studies; the MHC did not show significantly fewer or more amplicons that were either hypo- or hypermethylated. In general, the two studies drew the same three main conclusions. First, methylation levels are bimodally distributed (the majority of amplicons being either hypo- or relatively hypermethylated). Second, methylation profiles show tissue specificity. Third, many regions display interindividual variation.

Other projects have used chromatin immunoprecipitation (ChIP) coupled with next-generation sequencing for high-throughput profiling of histone changes and delineation of transcription factor binding across different cell types and cellular stimuli (Barski *et al*., 2007; Mikkelsen *et al*., 2007). Developing epigenetic resources such as these will add another layer of information to genomic annotation, by not only defining regions that control gene activity in cell types, but also by uncovering sites of imprinting and allele-specific transcription. These findings will benefit our understanding of complexities of MHC gene function in normal cellular homeostasis and disease states. In this way, epigenetic research could provide markers for disease conditions and new targets for drug development. It may offer new explanations in well-studied areas such as autoimmunity, and will provide a basis for novel approaches to research on environmental effects, nutrition and ageing in complex disease. In this respect, the finding that DNA methylation profiles are associated with DNA alleles is a significant discovery (Raval *et al*., 2007), which could provide new insight into the rather inconsistent MHC association studies in complex disease. Epi-alleles and epihaplotypes that combine epigenetic information with DNA sequence could provide improved risk prediction for a disease than either of the two factors analysed alone. Two concepts have been proposed for integrating disease genetics and epigenetics: haplo-epitype (hepitype) analysis (Murrell *et al*., 2005) and CDGE (common disease genetic epidemiology in the context of both genetic and epigenetic) analysis (Bjornsson *et al*., 2004).

Finally, although the focus in this section has been on how epigenetics modifies gene activity, it is also of interest that chromatin modifications have been evoked as controlling activity of recombination and chromosome breakpoints (Neumann & Jeffreys, 2006; Barski *et al*., 2007), which could have significant bearing on the LD relationships within the MHC.

## Regulatory RNAs

It has recently come to light that a significant proportion of the genome expresses non-coding RNA, and that there is extensive overlap of transcriptional units and regulatory elements (Kapranov *et al*., 2007). Some of these abundant RNA transcripts, termed microRNA (miRNA), together with sequence polymorphisms and epigenetic factors, modulate gene expression at transcription and post-transcriptional level. The regulatory properties of miRNAs are implicated in gene coexpression, gene silencing, imprinting and DNA methylation, and are achieved by affecting mRNA degradation and translation. Interestingly, miRNAs often arise from demethylation of tandem repeats (Lippman & Martienssen, 2004), and thus interindividual variability in tandem repeats could account for differences in gene expression that result in inherited phenotypic variability (Flanagan *et al*., 2006).

The emerging paradigm that genomic architecture is not co-linear, but is instead interleaved and modular, has clear repercussions in MHC research in terms of increased information content, transcriptional complexity, regulation of immune functioning and evolution. The involvement of regulatory RNA in disease predisposition clearly warrants more detailed investigation along with increased scrutiny and consideration of non-coding DNA sequences.

Intriguingly, miRNAs may also function in interspecies regulation involving viral miRNAs and host immune system genes. A recent study shows that a miRNA encoded by human cytomegalovirus (HCMV) during infection reduces host cell killing by NK cells by specifically down-regulating expression of a ‘stress-induced’ gene (*MICB*) that encodes a ligand for the NK cell activating receptor NKG2D (Stern-Ginossar *et al*., 2007). The miRNA-based immunoevasion mechanism, exploited by HCMV, adds to the many ways infectious agents have of immune evasion (Wu *et al*., 2003; Wang *et al*., 2005; Pennini *et al*., 2006). One can envisage ‘counteractive’ evolution of the host immune defence genes in order to disrupt the interaction of pathogen miRNA with target host genes.

As a final point, down-regulation of host genes by interaction of their mRNAs with viral miRNA could lead to novel therapeutics. Targeting these viral miRNAs might constitute an antiviral therapy, while mimicking their role could provide a means of immunosuppression.

**Figure 7:**
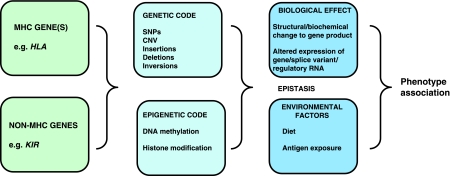
A summary of potential factors involved in an MHC association with a complex disease. CNV, copy number variation; HLA, human leucocyte antigen; KIR, killer immunoglobulin receptor; SNP, single-nucleotide polymorphism.

A diagrammatic representation of factors discussed in this review that may combine to influence MHC associations with complex disease phenotypes is given in Fig. 7.

## Conclusion and outlook

The MHC provides a prototype for the genetic footprints created by clusters of genes that are individually under balancing selection and whose products functionally interact. Epistasis may lie behind some aspects of the MHC, such as ancient highly diverged haplotypes that seem to be evolving independently except for ‘block shuffling’. It could also be responsible for difficulties in locating single gene contributions to disease in which multiple-linked interacting genes are at work. Methods of analysis that allow for, or exploit, the phenomenon of epistasis are being developed and successfully applied. Allowing for epistatic interactions may permit identification of genetic effects that might otherwise have remained undetected.

The index of variation built from the MHC Haplotype Project and related projects have dramatically improved our ability to define the sequence variants within the MHC involved in human disease. Higher-resolution analysis of gene CNV and structural variation associated with MHC genetic diversities in human populations promises to provide further insights into complex diseases and quantitative genetic traits. Technological developments will play a key role in future MHC studies. In the very near future, next-generation sequencing platforms are likely to identify new miRNAs and provide quantitative tag-based gene expression data relevant to disease phenotypes. Large-scale re-sequencing could potentially replace tag-based WGA studies, even though the WGA approach has become commonplace only relatively recently. However, high-throughput re-sequencing projects will bring new analytical challenges, especially in the detection of functionally important but rare variants among the background diversity.

Recent epigenetic studies provide a first impression of how the MHC is packaged and functionally organized to regulate gene activities. Clearly, we have much more to learn in mapping the MHC. It is already recognized that chromatin domains that envelop individual gene regulatory regions, whole genes and groups of genes modulate cellular activities. Perhaps a complete view of the MHC disease involvement will require maps of chromatin topography that characterizes the cell types involved in disease. The net impact will be a substantially enhanced annotation of the MHC, which will in turn dramatically increase our ability to interpret sequence-level variations.

The projects described here are the start of another long and fertile period of MHC research. Essentially, a truly integrated epigenetic–genetic approach to common disease is sought, including development of molecular technologies to identify and screen gene regulators and elucidate how and when transcription factors and chromatin remodelling proteins interact with the genetic code. Such study promises to unveil some of the mysteries that have long shrouded the MHC, and provide future directions for diagnostics and therapeutic interventions of many prevalent and debilitating diseases.
